# The effect of adjuvants on spray droplet size from hydraulic nozzles

**DOI:** 10.1002/ps.5742

**Published:** 2020-02-03

**Authors:** Rick Sijs, Daniel Bonn

**Affiliations:** ^1^ Van der Waals‐Zeeman Institute University of Amsterdam Amsterdam The Netherlands

**Keywords:** breakup, sprays, drops, adjuvants

## Abstract

**Background:**

When spraying simple liquids through a hydraulic nozzle, the mechanisms that affect breakup into droplets have recently been described. However, it is unknown how the droplet size distribution changes when surfactant‐based adjuvants are added to the spray.

**Results:**

When spraying different surfactant‐based solutions containing commercial adjuvants, the breakup of the different liquids behaves in the same way as for water‐only sprays, but the droplet sizes are smaller. By replacing the equilibrium surface tension with the dynamic surface tension at a surface age of ~20 ms, the volume mean droplet size variation with the Weber number, flow rate and nozzle geometry follows the predictions established for pure water sprays. When we rescale the droplet size distribution with the mean droplet size, all distributions collapse onto a single curve and can be described by a compound Gamma function.

**Conclusion:**

Addition of a number of surfactant‐based adjuvants to an agricultural spray is observed to lead to a slight decrease in the volume mean droplet size. We show that the effects of these adjuvants on the drop size can be understood by taking into account the nozzle geometry, the flow rate, the liquid and air densities and the dynamic surface tension of the surfactant solutions at a surface age of approximately 20 ms. © 2020 The Authors. *Pest Management Science* published by John Wiley & Sons Ltd on behalf of Society of Chemical Industry.

## Graphical abstract

Surfactant additives are often used for agricultural sprays; we show that this leads to smaller droplets due to the dynamic surface tension.

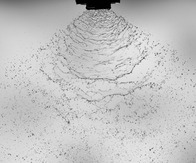

## INTRODUCTION

Spraying is a common everyday‐life process used in agriculture, printing, de‐icing, spray painting, firefighting, and drug administration,^1^, ^2^ In agricultural pesticide spraying it is important that drop sizes created when spraying through hydraulic nozzles are properly selected to ensure good deposition and coverage,^3^ but not too small to prevent environmental pollution because of airborne spray drift.^4^, ^5^, ^6^ A good understanding of what determines the drop size in agricultural sprays is important to achieve the most efficient way of spraying pesticides on plants while having as little as possible environmental pollution caused by spray drift. A recent paper of Kooij *et al*.^7^ describes for the first time how to predict the droplet size of simple water sprays for agricultural flat fan and hollow cone nozzles. They found that the volume mean droplet size can be predicted from the nozzle geometry, the flow rate and the surface tension of a fluid.

However, most sprays used in practical applications are not simple water sprays but commonly contain additives or adjuvants that can notably change the surface tension, which is important for the drop size. We can distinguish between formulation additives, which are non‐biologically effective compounds that are combined with the active ingredient in a formulation, and spray adjuvants, which are added to the tank containing the pesticide mixture.^8^ Here, we focus on the latter category.

Perhaps the most used agricultural spray adjuvants are based on surfactants that lower the surface tension.^9^ These are commonly used in the tank mix as wetting agents to enhance droplet spreading and sticking on target surfaces, as well as penetration into the leaf and barriers for active ingredient uptake, but they also can change the spray dynamics.^10^ Hilz and Vermeer^11^ reviewed spray additives in general and elucidate three adjuvant properties that influences the breakup of a liquid sheet: viscosity, surface tension and the presence of inhomogeneities such as emulsion droplets or solid particles. The results of Kooij *et al*.^7^ however appear to show that for the nozzles and pressures they use, the breakup is independent of viscosity. The same nozzles and pressures are used, and in addition the surfactant additives we use do not influence the viscosity very much. We also only consider homogeneous solutions (no emulsions, notably) so that the presence of heterogeneities can also be neglected here. This article is therefore limited to aqueous sprays containing surfactants; in this case the volume mean droplet size of a spray only depends on the surface tension of the fluid; generically a lower surface tension results in a decrease in the volume mean droplet size^7^, ^12^ because it becomes easier to make small drops when the surface tension is lower.^7^ However the important point is that when surfactant‐based adjuvants are sprayed, the surface tension of the droplets is not at equilibrium, but changes in time as surfactant molecules migrate to the surfaces of freshly formed droplets.^13^ A decrease in dynamic surface tension generally results in a larger fraction of small droplets and a decrease in average droplet size.^14^, ^15^ Other studies found that, because of the relatively slow dynamics of surfactant molecules, they do not influence the droplet size distribution.^7^ Ellis and Tuck^16^ also found that the effect on droplet size differs between different nozzles and for different types of surfactants. The review of Spanoghe *et al*.^8^ summarizes a vast body of research on sprays with surfactants, but their conclusions do not allow to predict the effect of surfactants on e.g. the drop size. This is what we aim at doing here: by focusing on ‘simple’ surfactants (and not complicated formulations) we aim at developing a more complete understanding of the influence of surfactant‐based adjuvants to sprays and how to predict the volume mean droplet size.

The recent paper of Kooij *et al*.^7^ described for the first time how to predict the volume mean drop size of simple sprays of water using agricultural nozzles. The present article is a first follow‐up and describes how agricultural adjuvants based on surfactants affect the droplet size distribution of sprays and how to predict the volume mean drop size. Different surfactant‐based adjuvants are sprayed, using simple, agricultural flat fan and hollow cone nozzles at various pressures, and the droplet size distribution is measured. To have a complete data set on at least one typical adjuvant, we sprayed solutions of Agral Gold, and sprayed this with different concentrations, nozzles and pressures. Furthermore, we sprayed different other adjuvants only at the label dosage. The choice of using only relatively simple soluble surfactants limits the scope of the current investigation; there are of course different spray additives, for example polymer solutions,^17^ emulsions,^18^ etc., and many other types of sprays and nozzles (air induced nozzles, pre‐orifice nozzles, atomizers, etc.) but these are beyond the scope of the present article.

## MATERIALS AND METHOD

### Spray adjuvants

**Figure 1:**
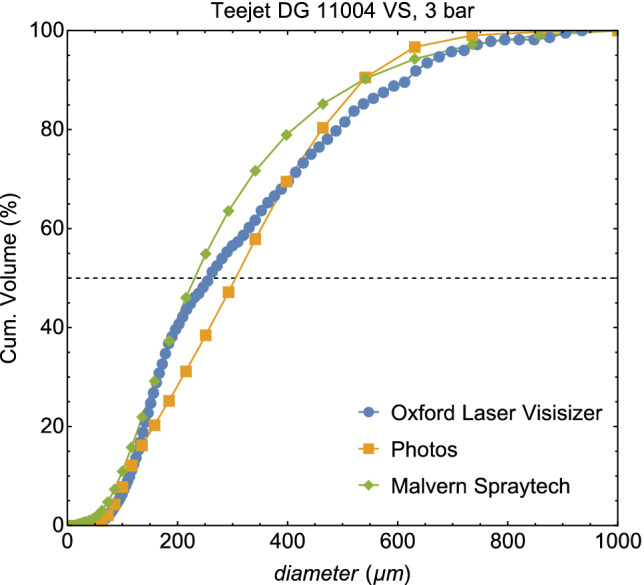
The comparison between the Malvern Spraytech, photographs and the Oxford Laser Visisizer.

The breakup of sprays with adjuvants for flat and conical hydraulic nozzles was studied. A flat fan nozzle produces a flat liquid sheet, where the conical nozzle produces a circular sheet or cone‐shaped spray that can be solid or hollow depending on the internal geometry of the nozzle. The Malvern Spraytech is used (Malvern, UK) for determining the droplet size distribution, a method based on laser diffraction. When the laser encounters a droplet, part of the laser energy will be reflected, another part of the energy diffracted and the last part absorbed. The diffraction angle is inversely proportional to the size of the droplet, so the light diffraction pattern allows us to obtain the droplet size distribution by assuming a spherical shape for the droplets.^19^, ^20^ We measured 40 cm below the nozzle, where, for all nozzles, pressures and fluid parameters used in this study, no further breakup occurs. We also measured the droplet size distribution with an Oxford laser Visisizer, an image‐based technique, and by making photographs with a short light pulse and a high‐resolution Nikon camera. All these techniques are compared with each other and in Fig. 1 it can be observed that the results from the different measuring techniques are comparable. A more detailed comparison for different nozzles and spraying pressures is beyond the scope of the current article, and will be published elsewhere.

**Figure 2:**
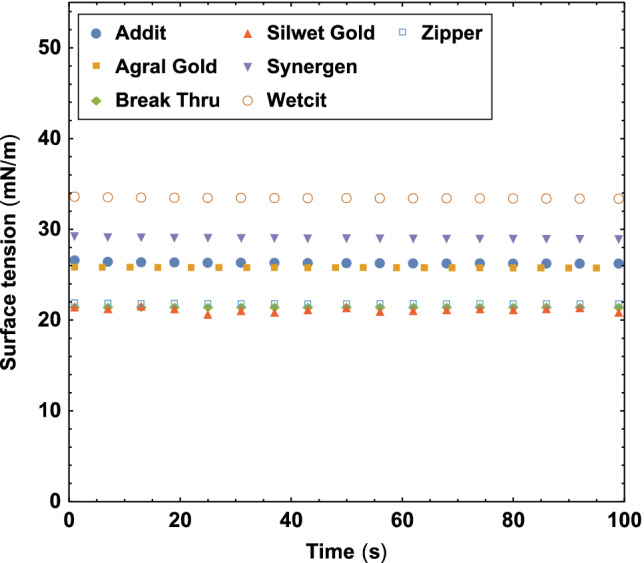
Equilibrium surface tension as a function of time.

**Figure 3:**
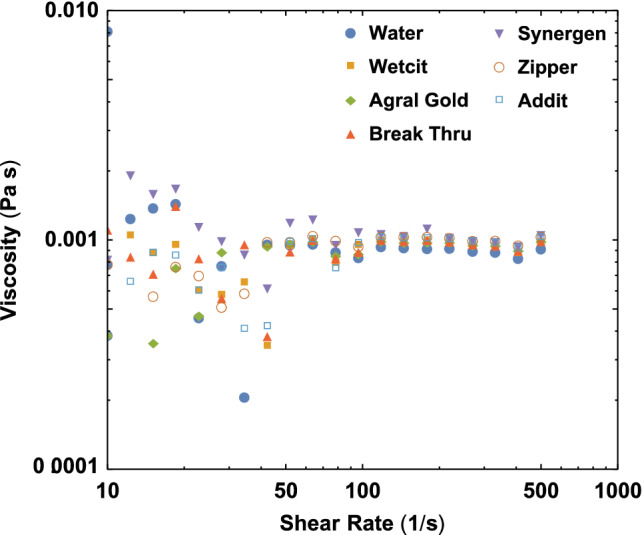
The rheology properties of the different adjuvants.

Different commercial adjuvants, see Table 1, were sprayed with flat fan and conical nozzles at different operating pressures between 1 and 5 bar, see Table 2. The equilibrium surface tension of the adjuvants was measured using the Krüss Force tensiometer with the Wilhelmy plate method, see Fig. 2. Table 3 describes the used nozzles, specifying their opening areas and discharge coefficients, which account for losses in the flow rate. The used nozzles are the same as those used by Kooij *et al*.^7^ Figure 3 shows the rheology properties of all adjuvants, measured with an Anton Paar MCR 502 Rheometer using a 50 mm Cone‐Plate geometry. The noise at low shear rate comes from the limited torque resolution of the rheometer. It can be seen that the surfactant‐based adjuvants are indistinguishable from pure water to within the experimental accuracy.

**Table 1:** Spray adjuvants and their data used in this study

Adjuvant	Manufacturer	Chemical composition	*σ*_equilibrium_ (mN/m)	*σ*_dynamic_ at 15 ms (mN/m)	σ_dynamic_ at 20 ms (mN/m)
Addit	Koppert	Polyether trisiloxane	26.4	68	68
Agral Gold	Modify	AOT (di‐octyl sulfosuccinate)	25.8	71	70
Agral Gold			25.8	56	53
Agral Gold			25.8	47	44
Break Thru S240	Evonik	Trisiloxane	21.6	47	43
Silwet Gold	Arysta	Oxirane, methyl‐, polymer with oxirane, mono[3‐[1,3,3,3‐tetramethyl‐1‐[(trimethylsilyl)oxy]disiloxanyl]propyl] ether	21.3	64	63
Synergen	Clariant	*n*‐Methyl‐alkyl glucamide	29.1	50	49
Wetcit	Oro Agri	Alcohol, C12–14, ethoxylated, sulfates, sodium salts	33.5	65	63
Zipper	Modify	Polyether modified polysiloxane	21.8	64	61
Zipper			21.8	64	61

The equilibrium surface tensions were measured with a Krüss Force tensiometer, using the Wilhelmy plate method. Dynamical surface tension values at *t* = 20 ms were determined using a Krüss Bubble Pressure Tensiometer BP50 followed by fitting to Eqn (1).

**Table 2:** Spray adjuvants and the spray parameters used in this study

Surfactant	Concentratoion (%)	Spray nozzle	Spray pressure (bar)
Addit	0.5	Teejet 11003	3
Agral Gold	0.04	All nozzles	1–5
Agral Gold	0.18	All nozzles	1–5
Agral Gold	0.36	All nozzles	1–5
Break Thru S240	0.5	Teejet 11002	3
Silwet Gold	0.1	Teejet 11003	3
Synergen	0.5	Teejet 11003	3
Wetcit	0.5	Teejet 11003	3
Zipper	0.1	Albuz ATR 80	2–5
Zipper	0.1	Teejet 11002, 11003, 11004	1–5

**Table 3:** Nozzles used in this study

Nozzle	Type	Area (m^2^)	Discharge coefficient
Teejet XR 11002	Flat	5.2 × 10^−7^	0.94
Teejet XR 11003	Flat	8.3 × 10^−7^	0.94
Teejet XR 11004	Flat	1.1 × 10^−6^	0.91
Albuz API 11003	Flat	8.8 × 10^−7^	0.85
Albuz ATR 80	Cone	1.1 × 10^−6^	0.34

The discharge coefficients account for losses in the flow rate. XR, API and ATR stand for the type of the nozzle.

### Dynamic surface tension measurement

As the surface tension of the droplets is a dynamic parameter, we measured the dynamic surface tension at surface ages between 15 and 10 000 ms using a Krüss Bubble Pressure Tensiometer BP50 (KRÜSS GmbH, Hamburg, Germany). These data are subsequently fitted with the equation of Hua and Rosen:^21^

(1) $$\sigma \left(t\right)=\sigma_{\infty }+\;\frac{\sigma_{0}-\sigma_{\infty }}{1+{\left(\frac{t}{\tau }\right)}^{n}}$$

**Figure 4:**
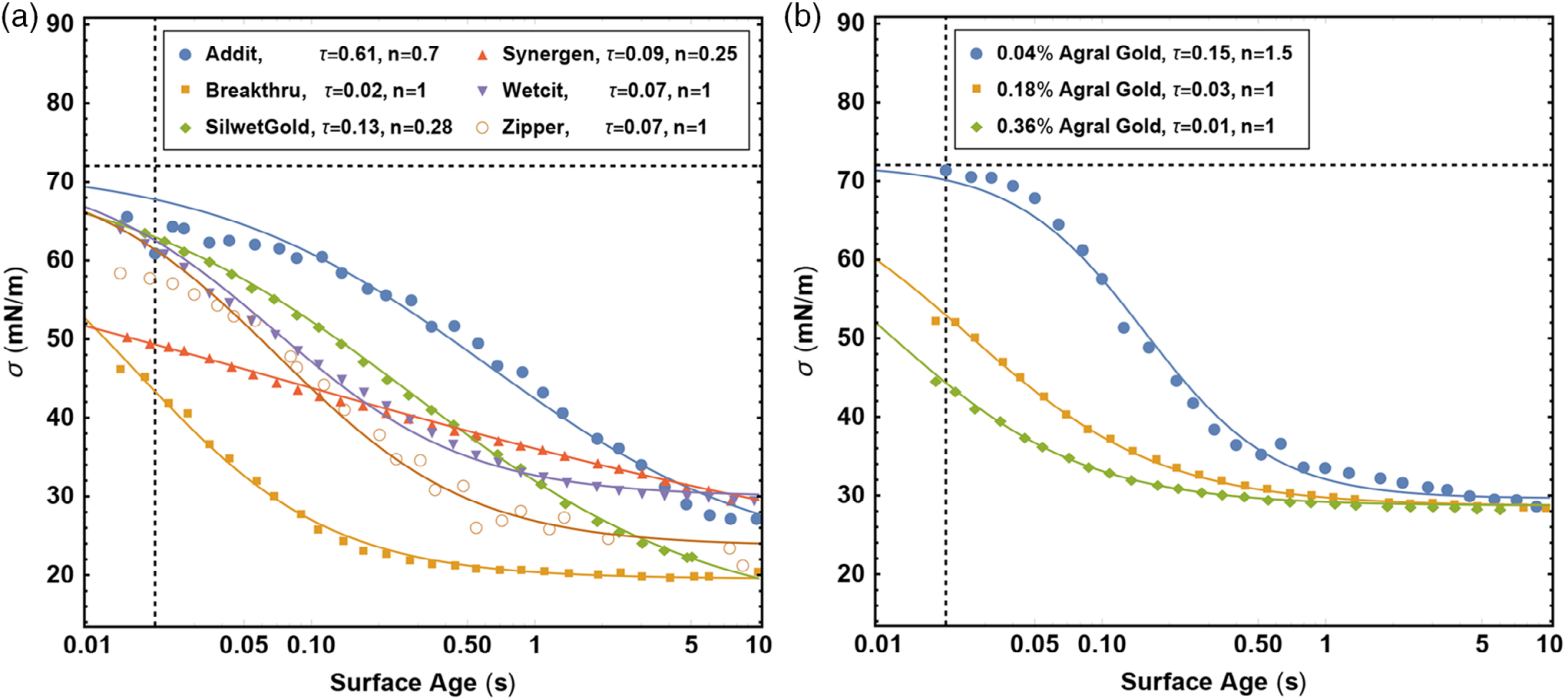
Surface tension as a function of time for six adjuvants (a) and for three different concentrations of adjuvant Agral Gold (b). Solid lines are fits to Eqn (1) with fitting parameters indicated in the legend; τ the characteristic time and *n* the power of *t* over *τ*. Vertical dotted lines indicate *t* = 20 ms, the breakup time of a sheet, and the vertical dotted lines indicated the surface tension of water, 72 mN/m.

Here, *σ*_∞_ is the equilibrium surface tension, *σ*_0_ the surface tension of water, 72 mN/m, τ a characteristic time and *n* ≈ 1 for surfactants.^22^ As shown in Fig. 4, the data correspond well to Eqn (1) with characteristic times between 0.01 and 0.61 s and *n* between 0.25 and 1.5.

## RESULTS AND DISCUSSION

### Droplet formation

**Figure 5:**
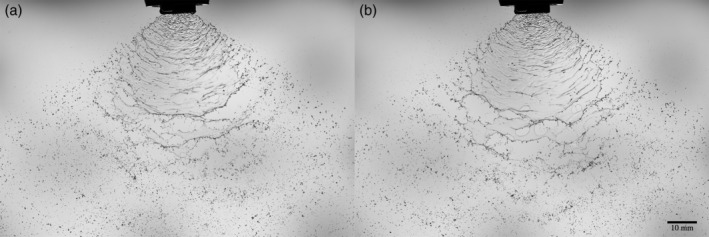
High‐speed photographs of (a) water and (b) water + 0.1% Zipper for a Teejet XR 11004 VK nozzle at 2 bar. The flapping and breakup process are clearly visible in both cases.

Figure 5 shows typical examples of the breakup of a liquid sheet, with and without adjuvants, as observed by high‐speed photography. Waves on the surface, produced by friction with the surrounding air, is the main breakup mechanism for agricultural spray nozzles.^23^ These waves grow in amplitude, causing modulations of the sheet. The modulations will cause the sheet to become so thin that it will rupture, creating sheet fragments of a well‐defined size, the Squire wavelength. These fragments will contract to form ligaments due to the Rayleigh–Taylor instability, the internal instability of the sheet accelerated perpendicular to the plane of the liquid sheet.^24^ The local diameters of the ligaments decrease until they break into droplets; this happens because of the Rayleigh‐Plateau instability that is due to the surface tension between the liquid and the air.^25^, ^26^, ^27^ Figure 5 reveals that, at least qualitatively, the breakup process happens in the same way for water and for the adjuvant solution.

### Volume mean droplet size

Kooij *et al*.^7^ describe the final drop size for sprays on the basis of the diameter of the ligaments by considering mass conservation. The volume mean diameter (*D*_50_), the most common way to characterize droplet size, can subsequently be determined as follows:^7^

(2) $$D_{50}=\mathrm{Cb}\alpha^{-1/6}{\text{We}}^{-1/3}$$

Here, the density ratio *α = ρ*_air_ */ρ*_liquid_, the Weber number, *We = ρ*_liquid_ *v* ^*2*^ *b/σ*, *σ* the surface tension, *v* the liquid velocity (determined by the pressure), *b* the characteristic length (the minor axis of the elliptical opening of the nozzle), and *C* a constant of order unity. This equation allows us to determine the volume mean droplet size for at least pure water sprays.^7^

**Figure 6:**
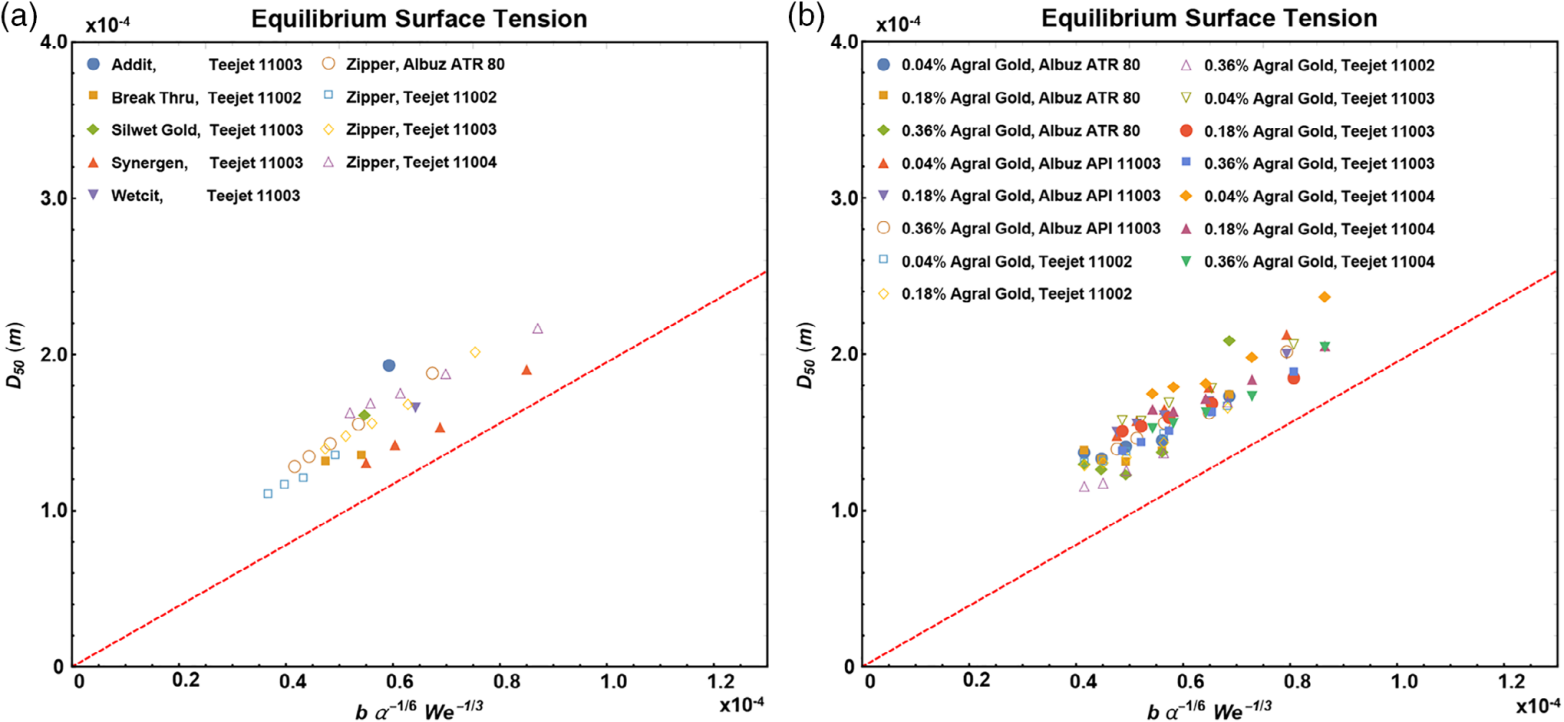
Volume mean diameter *D*_50_ plotted with Eqn (2) for different adjuvants and different nozzles (a) and for Agral Gold adjuvant at different concentrations and different nozzles (b). To calculate the Weber number, we used the equilibrium surface tensions listed in the legends. Red dotted lines have slope 1.95. Further details of the adjuvants and nozzles can be found in Tables 1, 2, 3.

Figure 6 compares the experimentally determined volume mean droplet sizes of the surfactant‐based adjuvants with Eqn (2) where the Weber number was calculated using the equilibrium surface tension. It can be seen that all data are parallel to the slope *C* = 1.95 (red dotted lines), only shifted to the left.

This shift points toward an error in the calculation of Eqn (2). Since *b* and *α* are constants, this error must come from determining the Weber number and hence the surface tension. While using the equilibrium surface tension in our calculations, it is well known that the surface tension is a dynamical parameter and changes significantly over the course of droplet breakup. During spraying, fluids experience high deformation rate flows as droplets break up and new surfaces are created rapidly.^13^ Figure 4 shows measurements of the dynamic surface tension at surface ages between 15 and 10 000 ms, revealing large variations. The question arises: how rapidly do the surfactants move towards the newly created surfaces and what is the typical surface age in the spraying process?

**Figure 7:**
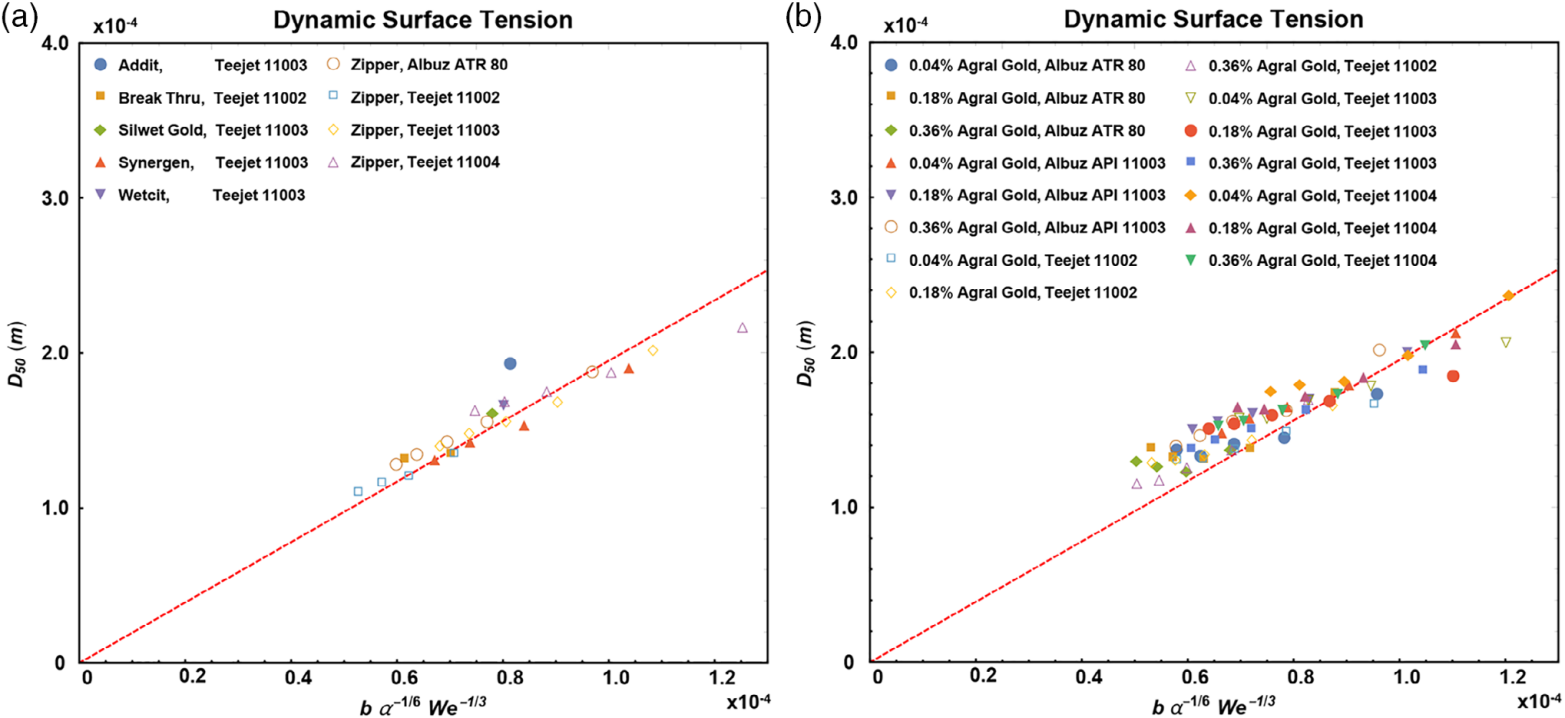
Volume mean diameter *D*_50_ plotted with Eqn (2) for different adjuvants and different nozzles (a) and for Agral Gold at different concentrations and different nozzles (b). To calculate the Weber number, we used the value of the dynamic surface tension at 20 ms extracted from fits such as shown in Fig. 4. Red dotted lines have slope 1.95. Further details of the adjuvants and nozzles can be found in Tables 1, 2, 3.

There are therefore two different timescales that need to be considered. The first is the characteristic time of the surfactant adsorption onto the surface. From Fig. 4 we see that the typical timescale for surfactant adsorption varies between roughly 100 and 1000 ms, depending on the type of surfactant and its bulk concentration. The second characteristic time is the characteristic time for the creation of new surfaces; we can take this to be the time after which the sheet breaks up after leaving the nozzle. The length after which breakup occurs under the nozzle is approximately 5 cm (Fig. 5) and the velocity of the liquid is about 10 m/s, which gives a breakup time around 5 ms in our sprays. There is at the moment no detailed theoretical understanding on how these two competing processes compete to give the dynamic surface tension during the breakup.^28^, ^29^ A detailed discussion can be found in Rozhkov *et al*.^30^ who also underline the differences in timescales between the dynamic surface tension and the breakup process. To compare with the model of Kooij *et al*.,^7^ we therefore use the experimental observations of Hewitt *et al*.^31^ who suggest a timescale of 20 ms for the dynamics of surfactant action during spraying and Battal *et al*.^32^ who found a characteristic time of about 15 ms. These observations suggest that the timescale is intermediate between that of surfactant adsorption and surface creation, which seems reasonable. We have previously shown that if we use a characteristic time of ~20 ms, and use the dynamic surface tension at that time to re‐calculate the Weber numbers, we find a satisfactory data collapse.^33^ This also holds here, as shown in Fig. 7. For our purposes, the difference between the dynamic surface tension at 15 ms or at 20 ms can be neglected, as can be observed in Table 1.

Table 4 gives the statistical comparison of the slopes for the equilibrium surface tension and the dynamic surface tension at 20 ms. It can be seen that the fit when using the dynamic surface tension is more accurate and the slopes are comparable as for simple water sprays as described by Kooij *et al*.^7^

**Table 4:** A statistical comparison of the slopes with the adjusted *R* ^2^ of the linear fit through the origin

	Slope different surfactants	Adjusted *R* ^2^ different surfactants	Slope Agral Gold	Adjusted *R* ^2^ Agral Gold
Equilibrium surface tension	2.67	0.9885	2.69	0.9915
Dynamic surface tension at 20 ms	1.95	0.9940	2.04	0.9913

For this article we used concentrations that are commonly used in agricultural sprays. Sijs *et al*.^33^ described in their paper that also for higher concentrations, up to ten times the critical micelle concentration (CMC), the breakup still happens in the same way as for the concentrations used in this article.

### Droplet size distributions

**Figure 8:**
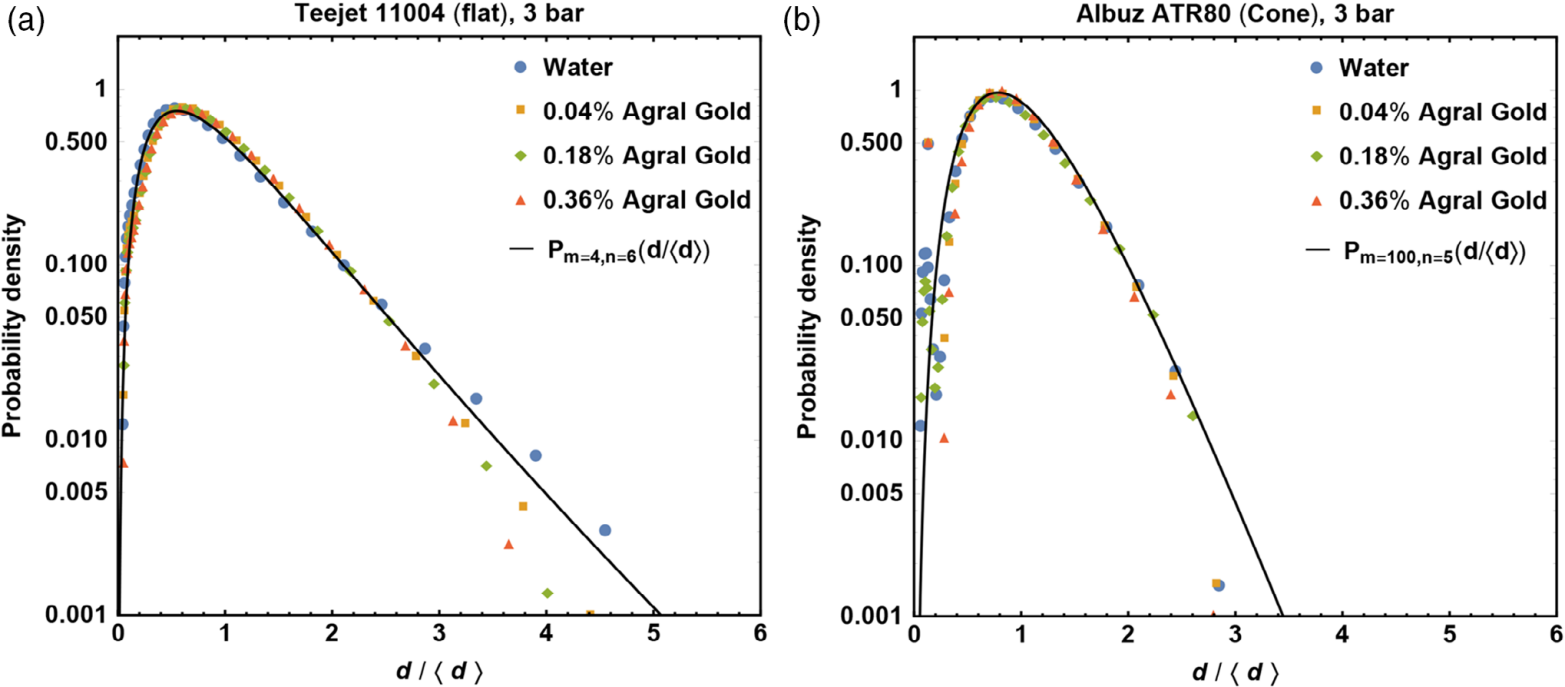
Normalized droplet size distribution, with on the *x*‐axis the droplet diameter rescaled with the mean droplet size, for different concentrations of adjuvant Agral Gold sprayed with the Teejet XR 11004 flat nozzle (a) and the Albuz ATR 80 conical nozzle (b) at 3 bar. The black lines are fits of the compound Gamma function (Eqn (3)).

Thus far we have focused on the average droplet size. However, the exact distribution of droplet sizes is also important for various applications. In Fig. 8 we plot the droplet sizes scaled to the mean value for different concentrations of adjuvant Agral Gold and two types of spray nozzles. The rescaling results in all data collapsing onto two nozzle‐dependent curves.

Villermaux^25^ has emphasized that the Gamma distribution best describes the droplet size distribution after breakup. With breakup of a liquid sheet, ligaments with different diameters grow. As they are also corrugated, the distribution can best be described by a two‐parameter compound Gamma function:^34^

(3) $$P_{m,n}\;\left(x=\frac{d}{<d>}\right)=\frac{2{\left(\mathrm{mn}\right)}^{\frac{m+n}{2}}x^{\frac{m+n}{2}-1}}{\Gamma \left(m\right)\Gamma \left(n\right)}\kappa_{m-n}\left(\;2\sqrt{\mathrm{mnx}}\;\right),$$

with *κ* the modified Bessel function of the second kind, parameter *m* setting the order of the ligament size distribution, and *n* the ligament corrugation, as described by Kooij *et al*.^7^ This compound Gamma function describes the droplet size distribution better than the log‐normal distribution, discussed by Kooij *et al*.^7^ for simple water sprays and by Sijs *et al*.^33^ for surfactants‐sprays. In Fig. 8 we show that the compound Gamma function describes the data very well, with the main difference between the two types of nozzles being the value of typical *m* = 4 for the flat fan nozzle, which implies that there is a broad range of ligament sizes, as the conical nozzle, with typical *m* = 100, gives ligaments with very similar sizes. The parameter *n* is low, *n* = 6 and *n* = 5 for respectively the flat and conical nozzle, which means that the ligaments are very corrugated.

**Figure 9:**
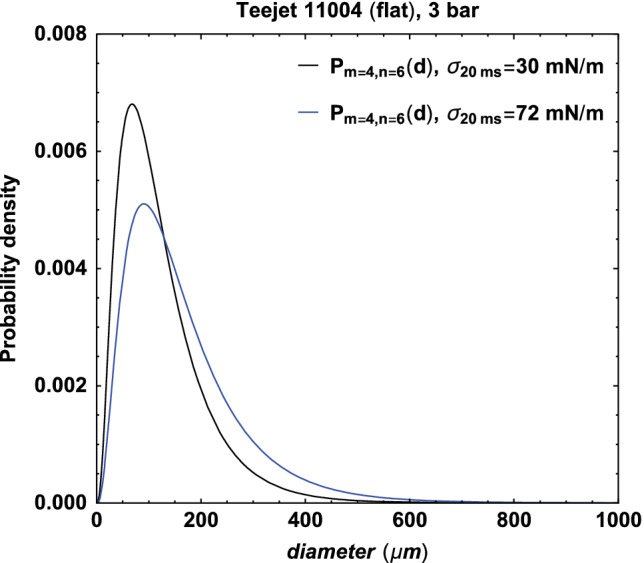
The droplet size distribution for the examples of spraying liquids with dynamic surface tension of 30 mN/m and 72 mN/m (water) at 20 ms with the Teejet 11 004 flat fan nozzle at 4 bar.

Now we are able to predict the volume mean droplet size and the typical droplet size distribution for surfactant‐based adjuvants when knowing the nozzle, the spraying pressure and the dynamic surface tension at 20 ms of the liquid. So if we for example take a Teejet 11 004 flat fan nozzle at an operating pressure of 4 bar with a liquid that has a dynamic surface tension of 30 mN/m at 20 ms and as a second example we spray water through the same nozzle with the same pressure, we are able to predict the droplet size. From the Teejet 11 004 nozzle we know that the opening area is 1.1 × 10^−6^ m^2^ with a discharge coefficient of 0.91 and the flow *q* is 1.82 L/min, or 3.03 × 10^−5^ m^3^/s. The volume mean droplet size can then be calculated from Eqn (2) and will be 123 μm and 164 μm for respectively the first (30 mN/m) and second (water, 72 mN/m) example. Figure 9 gives the droplet size distributions of these two examples with the compound Gamma function with the typical *m* = 4 and *n* = 6 for flat fan nozzles.

## CONCLUSION

The spray behavior of simple water sprays is studied by Kooij *et al*.^7^ They found a formula to predict the volume mean droplet size. We have studied a next step of predicting the volume mean droplet size of another spray; the spray behavior of solutions containing surfactant‐based adjuvants, as this is a common scenario for a wide range of real‐world applications. Comparing this behavior to what is established for pure water sprays, we find that, at least qualitatively, the process by which droplets are produced is the same. However, the methodology to predict the volume mean droplet size, as proposed by Kooij *et al*.^7^ for pure water sprays (Eqn (2)), fails, with the data appearing to be shifted. We explain this shift by considering the time‐dependency of the surface tension. During droplet breakup, new surface area is created rapidly. The surfactant molecules need time to move to the newly created surface, resulting in a time‐dependent surface tension. Assuming the surface has an ‘age’ of 20 ms at the moment of droplet breakup, as suggested by Hewitt *et al*.,^31^ and replacing the equilibrium surface tension in Kooijʼs analysis with the dynamic surface tension at the time of breakup, Eqn (2) is found to predict the volume mean droplet size of our tested range of adjuvants, concentrations and types of spray nozzles. Using the mean droplet size to rescale the droplet size distributions, we furthermore find that the data for different adjuvant concentrations collapse onto a single nozzle‐dependent curve and can be described by a compound Gamma function.

By establishing that the analysis used for pure water sprays also applies to sprays containing adjuvants but not any active ingredients. Provided the dynamic nature of the surface tension is taken into account, we conclude that for the tested surfactant‐based adjuvants, that decrease the surface tension by definition, also slightly decrease the volume mean droplet size.
